# The Association of Interstitial Fibrosis and Ki67 Marker Expression in Papillary Thyroid Carcinoma with Histopathological Findings and Prognosis of Total Thyroidectomy

**DOI:** 10.3390/jcm15145703

**Published:** 2026-07-21

**Authors:** Osman Anıl Savaş, Selçuk Mercan, Amir Mahdi Akbari, Mehrdad Sheikhvatan

**Affiliations:** Department of General Surgery, Faculty of Medicine, Istanbul Okan University, 34947 Tuzla, Istanbul, Turkey; osmananilsavas@gmail.com (O.A.S.); amirmahdiakbari937@gmail.com (A.M.A.)

**Keywords:** papillary thyroid carcinoma, prognosis, Ki67, interstitial fibrosis

## Abstract

**Background:** Interstitial fibrosis has been increasingly recognized as a histopathological feature with potential prognostic significance in papillary thyroid carcinoma (PTC). Ki67 expression has also been proposed as a prognostic marker in such patients. We investigated the extent of interstitial fibrosis and Ki67 expression in papillary thyroid carcinoma and its relationship with clinical and histopathological findings of the tumor, as well as prognosis of total thyroidectomy. **Methods:** This cross-sectional study was performed on 96 histological specimens obtained from patients with papillary thyroid carcinoma who had undergone total thyroidectomy. The extent of interstitial fibrosis of the tumor was examined in H&E-stained slides. The sections obtained were subjected to immunohistochemical staining with Ki67 monoclonal antibody to evaluate the expression of this marker. **Results:** Interstitial fibrosis did not have a significant relationship with tumor size, nor with the extent of tumor spread to surrounding tissues, as to lymph nodes or the vascular system. Percentage of interstitial fibrosis was not able to predict the outcomes after carcinoma surgery, such as patient mortality or tumor recurrence. A significant relationship was observed between Ki67 expression and involvement of the lymph nodes in the tumor area. The tracking of Ki67 expression could not predict the prognosis after surgery such as tumor recurrence or patient mortality. **Conclusions:** The expression of Ki67 is related to lymph node involvement in PTC. No significant relationship was observed between tumor biological characteristics and the percentage of interstitial fibrosis. The expression of these two markers cannot predict tumor recurrence and mortality.

## 1. Introduction

Differentiated thyroid cancer is the most common endocrine cancer in the world, with an annual incidence slightly higher in women than in men, and the fifth most common cancer in this sex, with a significantly increasing incidence [1,2]. The most common differentiated thyroid carcinoma is papillary carcinoma, which is responsible for more than 80% of thyroid cancers and originates from follicular cells [3]. With the advancement of imaging techniques, a significant increase in the incidence of papillary thyroid carcinoma has been observed since the 1990s [4]. Microscopically, the diagnosis of this tumor is made by observing papillary structures and some specific nuclear features, such as nuclear overlap, furrow formation, and intranuclear pseudoinclusions [5]. The prognosis of patients with papillary thyroid carcinoma is very good, with a 10-year survival rate of over 90%. These tumors sometimes show a very aggressive course [6].

Many clinical and pathological factors have been associated with poor prognosis in papillary thyroid carcinoma, including patient age and sex, tumor size, lymph node metastasis, capsular invasion, multicentricity, history of radiation, and spread to other tissues [6]. In addition, several histopathological features, such as extrathyroidal extension, vascular invasion, aggressive tumor variants, and incomplete surgical resection, have also been identified as important predictors of disease recurrence and reduced survival [7].

Interstitial fibrosis, composed of fibroblasts and varying amounts of collagen fibers, has been reported in a wide range of malignant tumors [8,9,10]. In papillary thyroid carcinoma (PTC), interstitial fibrosis represents an important clinicopathological feature and may aid in histopathological diagnosis [11]. Alterations within the tumor stroma can influence several aspects of tumor biology, including local invasion, metastatic potential, angiogenesis, and overall prognosis [12]. Previous studies have demonstrated that interstitial fibrosis is associated with an increased risk of recurrence and mortality in several malignancies [13,14,15]. Moreover, dense stromal fibrosis has been proposed as a useful diagnostic marker for PTC [16]. The prognostic significance of interstitial fibrosis in PTC remains incompletely understood, and the limited available studies have yielded inconsistent findings [17].

Cellular proliferative activity is also an important factor for evaluating the biological behavior of carcinomas. Currently, Ki67 is one of the most useful markers for evaluating cellular proliferative activity. This marker is commonly used to assess clinical progression and estimate the prognosis of malignant tumors [18]. Many studies have reported strong expression of Ki67 in many malignant tumors [19,20,21]. Ki67 has been proposed as a prognostic marker in prostate cancer [22]. In addition, measuring the intensity of Ki67 expression by immunohistochemical staining is a routine approach for prognostic evaluation in breast cancer [23]. However, there are still no consistent results regarding the use of Ki67 immunohistochemical staining in papillary thyroid carcinoma. One study showed no association between Ki67 expression and tumor size in papillary thyroid carcinoma [24], while other studies reported a positive correlation between Ki67 expression and the risk of extrathyroidal spread and metastasis [25,26].

Despite increasing interest in stromal remodeling and proliferative activity in papillary thyroid carcinoma, the prognostic significance of interstitial fibrosis and Ki67 expression remains incompletely understood. Previous studies have reported inconsistent findings regarding their association with aggressive histopathological features, recurrence, and survival, and few investigations have evaluated these markers together in patients treated with total thyroidectomy. Therefore, the present study was designed to address this gap by examining the extent of interstitial fibrosis and Ki67 expression in papillary thyroid carcinoma and determining their relationship with clinicopathological characteristics and postoperative outcomes. We hypothesized that increased interstitial fibrosis and/or elevated Ki67 expression would be associated with more aggressive tumor features and less favorable prognosis. Thus, the present study was designed to investigate the extent of interstitial fibrosis and expression of the Ki67 marker in papillary thyroid carcinoma and its relationship with other clinical and histopathological findings of the tumor.

## 2. Materials and Methods

This cross-sectional study was performed on histological specimens obtained from patients with papillary thyroid carcinoma that underwent total thyroidectomy between 2022 and 2024. This study was conducted in accordance with the Declaration of Helsinki, and the protocol was approved by the Ethics Committee of Shahid Beheshti University of Medical Sciences. Since this study was conducted on archived histological samples of the patients, no additional costs or invasive procedures were imposed on the patients. The patients’ information was also recorded in a coded form without mentioning their personal details. Patients were excluded from this study if they met any of the following criteria: (1) incomplete or missing medical records regarding key clinical or pathological variables; (2) follow-up duration of less than 3 months after surgery; (3) perioperative mortality within 30 days of surgery (if this study focuses on long-term outcomes); (4) presence of concomitant malignancies affecting survival analysis; and (5) patients who received preoperative therapy that could significantly alter baseline histopathological assessment.

Given the retrospective nature of this study, the sample size was determined by the total number of eligible patients who underwent the specified surgical procedure during the defined study period. No formal a priori power calculation was performed due to the exploratory design and the rarity of the condition. Post hoc considerations indicate that the available cohort provides adequate descriptive power for estimating incidence rates and generating preliminary survival outcomes, although limited events restrict multivariable modeling.

**Figure 1:**
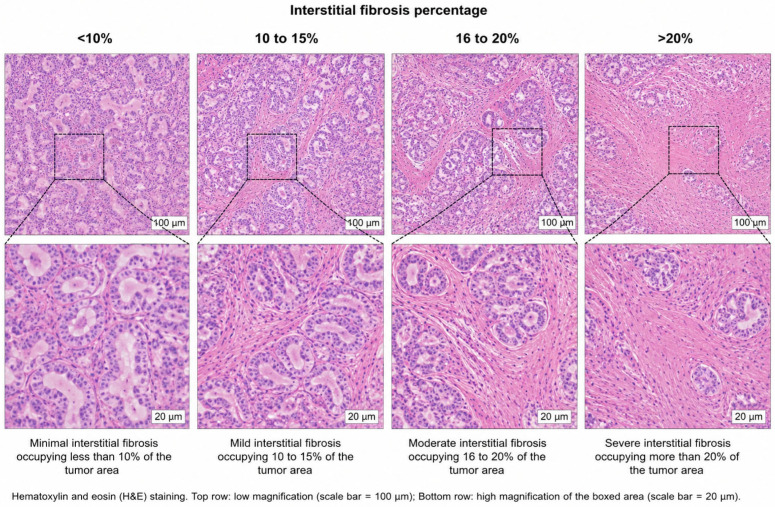
Representative histopathological images demonstrating different degrees of interstitial fibrosis in papillary thyroid carcinoma.

First, all hematoxylin and eosin (H&E)-stained slides from patients with papillary thyroid carcinoma were extracted from the pathology department archives of Okan hospital, Turkey. Patients whose clinical records were incomplete were excluded from this study. The extracted slides were reviewed by a pathologist and, in addition to confirming the diagnosis, histological subtype and other histopathological information including invasion into blood and lymphatic vessels, extrathyroidal extension, tumor focality, tumor size and lymph node involvement were recorded. Clinical information including age and sex of the patients was also extracted from their clinical records. In the next step, the degree of tumor interstitial fibrosis in the H&E-stained slides were examined. Accordingly, the samples were divided into four groups: no fibrosis/mild fibrosis, whose fibrosis area was lower than 10% of the total tumor, between 10 to 15% of the total tumor, between 16 to 20% of the total tumor, and more than 20% of the total tumor (Figure 1) [11].

For immunohistochemical analysis, the most representative formalin-fixed, paraffin-embedded (FFPE) tissue block containing adequate viable tumor tissue with minimal hemorrhage and necrosis was selected for each case. Serial 4 µm thick sections were cut using a microtome and mounted on positively charged slides. The sections were deparaffinized in xylene, rehydrated through graded alcohols, and subjected to heat-induced antigen retrieval according to the manufacturer’s protocol. Endogenous peroxidase activity was blocked prior to incubation with the primary antibody.

Immunohistochemical staining for Ki67 was performed on 4 μm thick formalin-fixed, paraffin-embedded tissue sections using a monoclonal anti-Ki67 antibody (clone MIB-1, Agilent Technologies/Dako, Glostrup, Denmark). Antigen retrieval was performed using citrate buffer (pH 6.0) in a heat-induced epitope retrieval system. After blocking endogenous peroxidase activity, sections were incubated with the primary antibody according to the manufacturer’s recommendations. Immunoreactivity was visualized using a horseradish peroxidase-based detection system and 3,3′-diaminobenzidine (DAB) chromogen, followed by hematoxylin counterstaining.

Ki67 expression was assessed independently by an experienced pathologist blinded to the clinicopathological data. Only distinct nuclear staining in tumor cells was considered positive. The hotspot method was used, whereby areas demonstrating the highest density of positively stained tumor nuclei were identified at low magnification. Subsequently, positive and negative tumor cells were counted in 10 high-power fields within these hotspot regions. Approximately 2000 tumor cells were evaluated for each case, and the Ki67 labeling index was calculated as the percentage of positively stained nuclei among all counted tumor cells.

Information regarding adjuvant treatment was collected from institutional oncology records, including radiotherapy, chemotherapy, or combined modalities administered postoperatively. The indication for adjuvant therapy was determined by a multidisciplinary tumor board based on tumor stage, histopathological features, and institutional guidelines. Patients were categorized according to whether they received adjuvant therapy (yes/no), and treatment modalities were further stratified when relevant for subgroup analysis. The timing, regimen type, and duration of therapy were also recorded when available.

SPSS version 27.0 statistical software was used to analyze the data obtained from this study. Quantitative data were expressed as mean ± standard deviation and qualitative data were expressed as frequency percentage. Each of the quantitative and qualitative data was statistically analyzed by appropriate statistical tests, including independent *t*-test and chi-square test. Also, the survival status and recurrence-free survival of the patients and its relationship with the degree of interstitial fibrosis and Ki67 expression were examined using Kaplan–Meier survival curve. The statistical significance criterion was considered to be *p* < 0.05.

## 3. Results

The baseline characteristics are summarized in Table 1. A total of 96 samples were studied. The mean age of the patients studied was 42.00 ± 12.77 years, ranging from 13 to 75 years, and 72.9% were women. In terms of the percentage of interstitial fibrosis, in 31.2% of cases, fibrosis was reported to be more than 20%. In terms of Ki67 marker expression, expression of 5% or more was reported in 12.5%. Regarding pathological behavior of the tumor, lymph node involvement was found in 45.8%, extranodal spreading in 21.9%, and lymphovascular invasion in 37.5%. Tumor recurrence was also revealed in 7.3%. Only 4.2% of the patients died in the follow-up. Regarding histopathological subtypes, the classic variant of PTC was the most frequently observed subtype, accounting for 71.9% of all cases. Papillary thyroid microcarcinoma of the classic subtype (microclassic variant) represented 20.8% of tumors, while the microfollicular variant was identified in 7.3% of patients. Thus, the vast majority of tumors exhibited classic papillary morphology, either as conventional PTC or as papillary microcarcinoma. In contrast, the microfollicular variant constituted only a small proportion of cases. Overall, classical histological patterns predominated in the study population, comprising more than 92% of all tumors, whereas follicular-patterned microcarcinomas were relatively uncommon.

**Figure 2:**
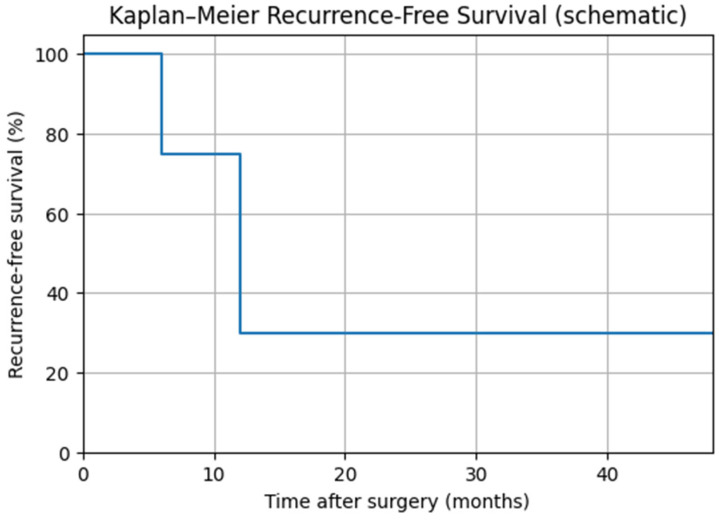
The Kaplan–Meier recurrence-free survival curve.

In total, tumor recurrence was reported in 7.3%, with a mean time interval between surgery and recurrence of 15.38 ± 14.64 months, ranging from 3 to 48 months. Mortality was also reported in 4.2%. Six-month and one-year recurrence-free survival in these patients was estimated to be 75% and 30% (Figure 2).

In terms of the relationship between Ki67 marker expression and baseline factors (Table 2), Ki67 expression was not associated with gender and age. There was no association of Ki67 expression with percentage of interstitial fibrosis, extrathyroidal spread of the tumor, extranodal spread, soft tissue involvement, and lymphovascular invasion. The mean tumor size in patients with and without Ki67 expression was 4.20 ± 3.13 cm and 1.95 ± 1.41 cm, respectively, indicating that the tumor size was higher in cases with Ki67 expression (*p*-value = 0.001). Also, in cases with and without lymph node involvement, the frequency of Ki67 expression was 22.7% and 3.8%, respectively, which was significantly higher in the first group (*p*-value = 0.005). The frequency of Ki67 expression in patients with and without tumor recurrence was 14.3% and 12.4%, respectively, with no significant difference (*p*-value = 0.882). Also, the frequency of Ki67 expression in deceased and living patients was 0.25% and 0.12%, respectively, with no significant difference (*p*-value = 0.440).

In terms of the relationship between the percentage of interstitial fibrosis with underlying and tumoral characteristics (Table 3), there was no association of interstitial fibrosis with gender, age, tumor size, extrathyroidal spread, soft tissue involvement, lymph node involvement, extranodal spread, lymphovascular involvement, or multifocal expansion. The frequency of tumor recurrence in the percentage of fibrosis below 10%, 10–15%, 15–20% and above 20% was 0.0, 12.8, 0.0 and 3.3%, respectively, indicating no relationship between the percentage of fibrosis and tumor recurrence (*p*-value = 0.233). Also, the frequency of mortality in the percentage of fibrosis below 10%, 10–15%, 15–20% and above 20% was 0.0, 4.3, 14.3 and 0.0%, respectively, indicating no relationship between the percentage of fibrosis and patient mortality (*p*-value = 0.164).

**Figure 3:**
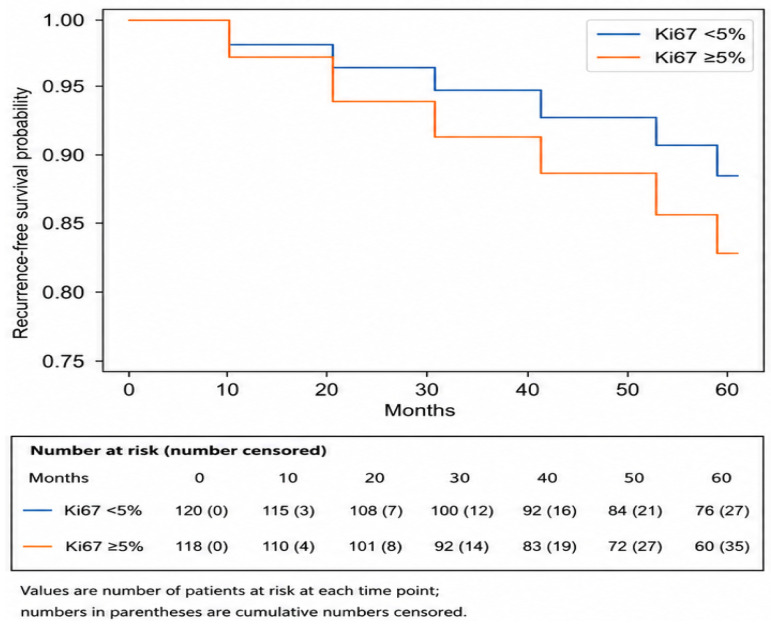
Kaplan–Meier analysis of recurrence-free survival (RFS) stratified by Ki67 expression (<5% versus ≥5%). The blue curve represents patients with Ki67 <5%, whereas the orange curve represents patients with Ki67 ≥5%. Survival probabilities are plotted over a 60-month follow-up period. The table beneath the graph displays the number of patients at risk in each subgroup at the specified time points on the x-axis. Numbers in parentheses indicate the cumulative number of censored observations up to each time point. Patients who remained recurrence-free at the end of follow-up or were lost to follow-up were treated as censored observations. The comparison demonstrates a trend toward lower recurrence-free survival among patients with higher Ki67 expression.

**Figure 4:**
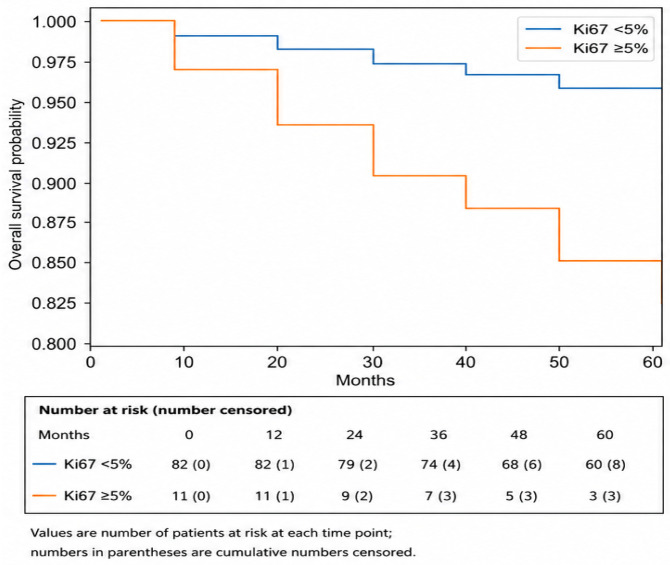
Kaplan–Meier analysis of overall survival (OS) stratified by Ki67 expression (<5% versus ≥5%). The blue curve corresponds to patients with Ki67 <5%, while the orange curve corresponds to patients with Ki67 ≥5%. Survival probabilities are presented over a follow-up period of 60 months. The accompanying risk table shows the number of patients remaining at risk in each subgroup at each time point displayed on the horizontal axis. Values in parentheses represent the cumulative number of censored patients. Censoring was applied to patients who were alive at the last follow-up or who were lost to follow-up. The survival curves indicate a tendency toward poorer overall survival among patients with elevated Ki67 expression.

Kaplan–Meier analyses demonstrated no significant differences in recurrence-free survival according to Ki67 expression status (<5% versus ≥5%; log-rank *p* = 0.68) (Figure 3). Likewise, overall survival did not differ significantly between patients with low and high Ki67 expression (log-rank *p* = 0.44) (Figure 4).

**Figure 5:**
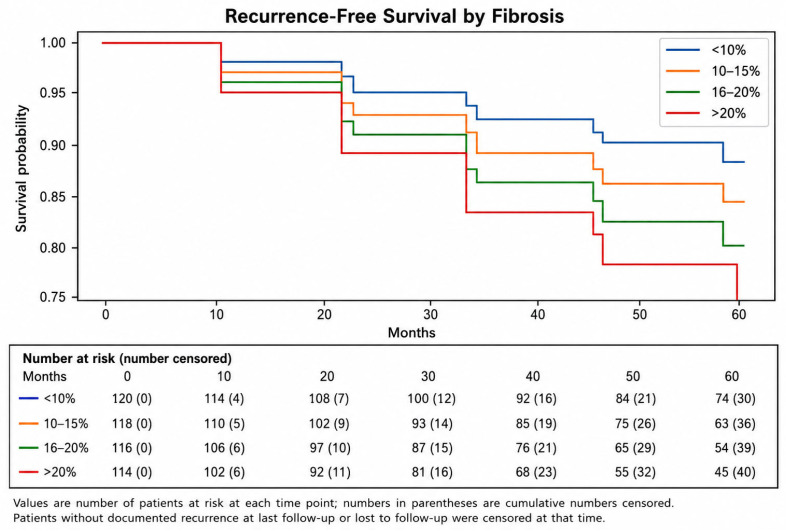
Kaplan–Meier curves illustrating recurrence-free survival (RFS) according to the degree of fibrosis within the tumor microenvironment. Patients were stratified into four groups based on fibrosis percentage: <10% (blue), 10–15% (orange), 16–20% (green), and >20% (red). Survival probabilities are presented over a 60-month follow-up period. The accompanying risk table beneath the graph displays the number of patients remaining at risk in each fibrosis subgroup at the corresponding time points shown on the x-axis. Numbers in parentheses indicate the cumulative number of censored observations up to each time point. Patients without documented recurrence at their last follow-up visit or who were lost to follow-up were treated as censored observations. The analysis demonstrates a progressive decline in recurrence-free survival with increasing fibrosis percentage, with patients exhibiting >20% fibrosis showing the least favorable outcomes during follow-up.

**Figure 6:**
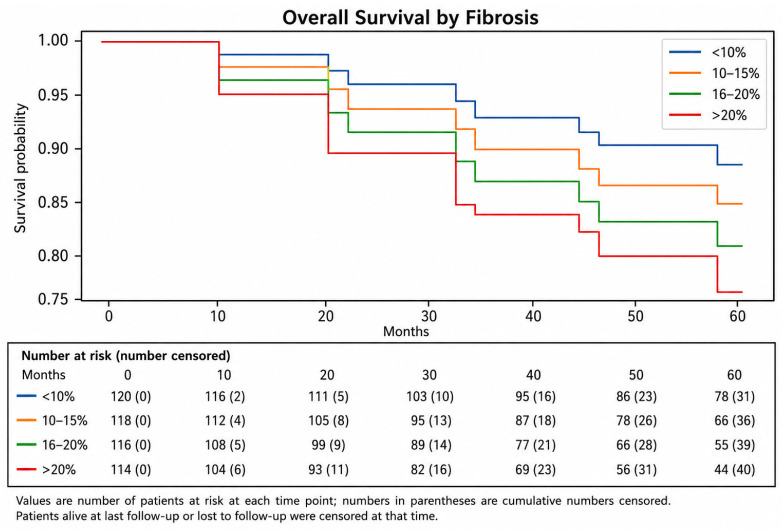
Kaplan–Meier analysis of overall survival (OS) stratified according to the degree of fibrosis within the tumor microenvironment. Patients were categorized into four fibrosis groups: <10% (blue), 10–15% (orange), 16–20% (green), and >20% (red). Overall survival probabilities were estimated over a follow-up period of 60 months. The table beneath the survival curves presents the number of patients at risk in each fibrosis subgroup at the corresponding time points shown on the x-axis, with values in parentheses indicating the cumulative number of censored observations. Patients who were alive at their last follow-up visit or who were lost to follow-up were treated as censored observations. A progressive reduction in overall survival was observed with increasing fibrosis percentage, with patients exhibiting >20% fibrosis demonstrating the poorest survival outcomes throughout the follow-up period. These findings suggest that extensive fibrosis may be associated with a less favorable prognosis.

When stratified according to interstitial fibrosis categories (<10%, 10–15%, 16–20%, and >20%), recurrence-free survival was comparable among groups (log-rank *p* = 0.27) (Figure 5). Similarly, no significant differences in overall survival were observed according to the extent of interstitial fibrosis (log-rank *p* = 0.19) (Figure 6). These findings support the results of the univariate analyses, indicating that neither Ki67 expression nor interstitial fibrosis was significantly associated with postoperative recurrence or mortality in this cohort.

## 4. Discussion

Interstitial fibrosis has been increasingly recognized as a histopathological feature with potential prognostic significance in PTC. The presence of stromal or interstitial fibrosis in the tumor microenvironment is thought to reflect tumor-stromal interactions that promote aggressive biological behavior. Studies have shown that PTCs with prominent fibrosis are often associated with larger tumor size, extrathyroidal extension, lymph node metastasis, and higher recurrence rates compared with nonfibrotic counterparts [14,15,16,17]. Fibrotic changes may reflect chronic inflammatory responses and epithelial-mesenchymal transition, both of which can enhance tumor invasion and metastatic potential. Therefore, assessment of interstitial fibrosis in the histopathological evaluation of PTC may provide additional prognostic information beyond traditional clinicopathological factors and could be useful for refining risk stratification and guiding postoperative management [18].

On the other hand, the proliferation index Ki67 serves as a valuable biomarker reflecting tumor cell proliferation and has important prognostic implications in PTC [19]. Increased Ki67 expression has been associated with aggressive tumor behavior, including larger tumor size, higher histological grade, extrathyroidal extension, lymph node metastasis, and an increased risk of recurrence [20,21]. Although PTC generally exhibits low proliferative activity, cases with high Ki67 labeling indices often show poorer clinical outcomes and shorter disease-free survival, suggesting that Ki67 can help identify patients at higher risk, despite poor histopathological features [22]. Consequently, the inclusion of Ki67 assessment in routine pathological evaluation of PTC may increase prognostic accuracy and support individualized treatment planning, especially in borderline or recurrent cases [23].

Accordingly, what was addressed in the present study was to investigate the extent of interstitial fibrosis and Ki67 marker expression in PTC and its relationship with other clinical and histopathological findings of the tumor. What we achieved as the first finding was that, firstly, in terms of the percentage of interstitial fibrosis in this carcinoma, only 5.2% of patients had interstitial fibrosis below 10%, while in 49.0% of cases, fibrosis was reported in the range of 10 to 15%, in 14.6% fibrosis in the range of 15 to 20%, and in 31.2% of cases, fibrosis was reported in more than 20%. Regarding the role of the assessment of the percentage of interstitial fibrosis in predicting the biological behavior and invasive characteristics of the tumor, the assessment of this characteristic did not have a significant relationship with tumor characteristics such as tumor size, the extent of tumor spread to surrounding tissues, as well as to lymph nodes or the vascular system. Also, the assessment of the percentage of interstitial fibrosis was unable to predict outcomes after carcinoma surgery, such as patient mortality or tumor recurrence. In the study by Can Sahin et al. in 2024 [24], interstitial fibrosis was significantly associated with bilaterality, multifocality, capsular invasion, and lymph node metastasis. Evaluation of tumor subtypes revealed a significantly increased risk of lymphovascular invasion in the follicular subtype [24]. In the 2022 Hong-Qun Wang study, the degree of fibrosis was associated with the proportion of papillary structure components. Univariate and multivariate survival analyses showed that recurrence-free survival (RFS) was longer in patients with moderate/severe interstitial fibrosis [25]. Also, in a 2018 study by Liu et al. [26], interstitial fibrosis was significantly associated with cytokeratin 19 and galectin-3. Analysis of patient outcomes showed that cancer-associated fibroblasts an independent prognostic factor for disease recurrence [26]. Interstitial fibrosis or desmoplastic stromal reaction has been increasingly reported as a histopathologic feature associated with more aggressive behavior in PTC. Early clinicopathologic studies showed that desmoplastic stromal reaction in papillary thyroid microcarcinomas was significantly correlated with aggressive features and a higher frequency of lymph node metastasis, suggesting that fibrosis may serve as a morphologic marker of invasion rather than a benign regenerative change [27]. Large clinicopathologic series focused on papillary thyroid microcarcinomas (PTMC) subsequently demonstrated that tumors with interstitial fibrosis had worse recurrence-free survival (e.g., one cohort reported a hazard ratio of ≈2.18 for recurrence in PTMC with fibrosis), suggesting a prognostic association beyond the simple presence/absence [28]. More recent multi-institutional and review data have reinforced the notion that fibrotic changes are associated with other unfavorable features such as multifocality, bilaterality, extrathyroidal extension, and lymph node disease, and that fibrosis—when quantified—is associated with parameters used in risk stratification [29]. Mechanistically, work from pathomics, single-cell, and tumor microenvironment studies suggests that activated stromal components (cancer-associated fibroblasts, extracellular matrix remodeling, TGF-β signaling, and immune-stromal crosstalk) provide a permissive site for epithelial-mesenchymal transition, invasion, and metastatic spread in PTC, providing a biological rationale for clinicopathologic associations [30]. Case series reporting of PTC with prominent fibromatous/desmoplastic stroma emphasize that when the stromal reaction is severe or distinct from a histological subtype, the clinical behavior can be unpredictable and sometimes more aggressive, including rare recurrences and distant spread [31]. In summary, the scientific literature is increasingly suggesting interstitial fibrosis as a marker—and possible mediator—of invasion and poorer prognosis in PTC subsets [32,33,34], but standardized assessment and higher-quality prospective data are needed before fibrosis can be accepted as a formal, independent prognostic variable.

According to the results of this study, firstly, expression of Ki67 below 5% was reported in 84 cases (87.5%) and expression of 5% or more was reported in 12 cases (12.5%). Second, among all the pathophysiological indicators of the tumor, a significant relationship was observed between Ki67 expression and regional lymph node involvement. The tracking Ki67 expression could not predict postoperative prognosis such as tumor recurrence or patient mortality. In the study by 34. Al-Timimi et al. in 2024 [35], disease-free survival (DFS) rates in PTCs differed significantly between patients with high and low Ki67 index. In a study by Lei et al. in 2023 [36], data analysis revealed a significant difference in Ki67 expression between patients and controls. Ki67 expression was not significantly associated with gender or age. In a study by Lindfors et al. in 2023 [37], male gender, tumor size >1 cm, multifocality, and Ki67 index >5% were independent risk factors for central compartment lymph node metastasis (CLNM). Ki67 is a nuclear antigen expressed during active phases of the cell cycle and is widely used as an indicator of proliferation in oncology. In PTC—typically a low-proliferative tumor—Ki67 has received attention because small subsets with higher labeling indices appear to exhibit more aggressive clinicopathological behavior. Therefore, numerous single-institution studies, cohort studies, and pooled analyses have evaluated whether Ki67 predicts invasive features (extrathyroidal extension, lymphovascular invasion, lymph node metastasis) and adverse outcomes (recurrence, disease-specific survival) [37].

Although the present study identified a significant association between Ki67 expression and lymph node involvement, neither Ki67 expression nor interstitial fibrosis was significantly associated with recurrence-free survival or overall survival. Therefore, these findings should be interpreted cautiously and viewed as exploratory rather than practice-changing. Nevertheless, the observed relationship between Ki67 expression and nodal disease suggests that proliferative activity may reflect certain aspects of tumor aggressiveness in papillary thyroid carcinoma. Additional studies with larger cohorts, longer follow-up, and greater numbers of outcome events are required to determine whether Ki67 expression or interstitial fibrosis can provide incremental prognostic information beyond established clinicopathological factors.

Several limitations should be considered when interpreting these findings. First, the retrospective single-center design may introduce selection bias and limits the generalizability of the results. Second, the relatively small sample size and the low number of recurrence (n = 7) and mortality (n = 4) events substantially reduced statistical power, particularly for survival analyses, and precluded multivariable modeling to adjust for potential confounders. Third, assessment of interstitial fibrosis was based on conventional histopathological estimation and Ki67 evaluation on immunohistochemical staining, both of which may be subject to sampling variability and observer-related bias. In addition, molecular alterations with known prognostic significance in papillary thyroid carcinoma, such as BRAF V600E and TERT promoter mutations, were not evaluated. These limitations may partly explain the differences between our findings and those reported in previous studies, and underscore the need for larger prospective multicenter investigations with standardized pathological assessment and longer follow-up.

## 5. Conclusions

In conclusion, the assessment of tumor proliferative activity and stromal alterations represents a promising approach for improving the pathological characterization of PTC. The integration of biomarkers reflecting both tumor biology and microenvironmental changes may contribute to a more comprehensive understanding of disease behavior and facilitate more refined risk stratification. Although the prognostic significance of these parameters requires further validation in larger prospective studies with longer follow-up periods, their evaluation may enhance current histopathological assessment and support the development of more individualized management strategies for patients with papillary thyroid carcinoma.

## Figures and Tables

**Table 1:** Baseline characteristics and tumor parameters.

Mean Age, Year	42.00 ± 12.77
Gender, %	
Male	26 (27.1)
Female	70 (72.9)
Interstitial fibrosis percentage	
Less than 10%	5 (5.2)
10 to 15%	47 (49.0)
16 to 20%	14 (14.6)
Higher than 20%	30 (31.2)
Ki67 expression	
Less than 5%	84 (87.5)
Higher than 5%	12 (12.5)
Extrathyroidal spreading	7 (7.3)
Soft tissue involvement	23 (24.0)
Lymph node involvement	44 (45.8)
Extranodal spreading	21 (21.9)
Lymphovascular invasion	36 (37.5)
Mean tumor size, cm	2.23 ± 1.85
Focality	
Single	60 (62.5)
Multiple	36 (37.5)
Tumor recurrence	7 (7.3)
Death	4 (4.2)
Variant	
Classic pattern	89 (92.7)
Follicular pattern	7 (7.3)

**Table 2:** The association of Ki67 expression and baseline parameters.

Parameter	Ki67 Expression	*p*-Value
Gender, %		0.602
Male (n = 26)	4 (15.4%)	
Female (n = 70)	8 (11.4%)	
Interstitial fibrosis percentage		0.591
Less than 10% (n = 5)	0 (0.0%)	
10 to 15% (n = 47)	6 (12.8%)	
16 to 20% (n = 14)	3 (21.4%)	
Higher than 20% (n = 30)	3 (10.0%)	
Extrathyroidal spread		0.182
Presence (n = 7)	2 (28.6%)	
Absence (n = 89)	10 (11.2%)	
Soft tissue involvement		0.416
Presence (n = 23)	4 (17.4%)	
Absence (n = 73)	8 (11.0%)	
Lymph node involvement		0.005
Presence (n = 44)	10 (22.7%)	
Absence (n = 51)	2 (3.8%)	
Lymphovascular invasion		0.339
Presence (n = 36)	6 (16.7%)	
Absence (n = 60)	6 (10.0%)	
Focality		0.339
Single (n = 60)	9 (15.0%)	
Multiple (n = 36)	3 (8.3%)	
Tumor recurrence		0.882
Presence (n = 7)	1 (14.3%)	
Absence (n = 89)	11 (12.4%)	
Death		0.128
Died (n = 4)	1 (25.0%)	
Survived (n = 92)	11 (12.0%)	
Variant		0.525
Classic pattern (n = 89)	14 (15.7%)	
Follicular pattern (n = 7)	0 (0.0%)	

Between-group comparisons were performed using the chi-square test or Fisher’s exact test.

**Table 3:** The association of interstitial fibrosis percentage and baseline parameters.

Parameter	<10% (n = 5)	10 to 15% (n = 47)	16 to 20% (n = 14)	>20% (n = 30)	*p*-Value
Mean age, year	37.80 ± 8.58	40.15 ± 13.26	47.50 ± 11.44	43.03 ± 12.72	0.177
Gender, %					0.985
Male	1 (20.0%)	13 (27.7%)	4 (28.6%)	8 (26.7%)	
Female	4 (80.0%)	34 (72.3%)	10 (71.4%)	22 (73.3%)	
Mean tumor size, cm	1.40 ± 1.75	2.53 ± 1.89	1.99 ± 1.34	2.01 ± 2.00	0.546
Extrathyroidal spreading	0 (0.0%)	2 (4.3%)	1 (7.1%)	4 (13.3%)	0.448
Soft tissue involvement	0 (0.0%)	12 (25.5%)	4 (28.6%)	4 (13.3%)	0.613
Lymph node involvement	1 (20.0%)	22 (46.8%)	6 (42.9%)	15 (50.0%)	0.654
Extranodal spreading	1 (20.0%)	10 (21.3%)	2 (14.3%)	8 (26.7%)	0.827
Lymphovascular invasion	1 (20.0%)	20 (42.6%)	4 (28.6%)	11 (36.7%)	0.648
Multifocal spreading	3 (60.0%)	17 (36.2%)	5 (35.7%)	11 (36.7%)	0.767
Tumor recurrence	0 (0.0%)	6 (12.8%)	0 (0.0%)	1 (3.3%)	0.233
Death	0 (0.0%)	2 (4.3%)	2 (14.3%)	0 (0.0%)	0.164
Variant					0.184
Classic pattern	4 (80.0%)	44 (93.6%)	13 (92.9%)	28 (93.4%)	
Follicular pattern	1 (20.0%)	3 (6.4%)	1 (7.1%)	2 (6.6%)	

Between-group comparisons were performed using the chi-square test for qualitative indices and ANOVA test or Kruskal–Wallis H test for quantitative indices.

## Data Availability

The data associated with this paper are available from the corresponding author upon reasonable request.

## Abbreviations

PTC: papillary thyroid carcinoma; H&E: hematoxylin and eosin; FFPE: formalin-fixed, paraffin-embedded; HPFs: high-power fields; RFS: recurrence-free survival; PTMC: papillary thyroid microcarcinomas; DFS: disease-free survival; CLNM: central compartment lymph node metastasis.
